# Determination of Phosphodiesterase Type-5 Inhibitors (PDE-5) in Dietary Supplements

**DOI:** 10.3390/molecules28104116

**Published:** 2023-05-16

**Authors:** Oana Ramona Cătălina Gheorghiu, Anne Marie Ciobanu, Claudia Maria Guțu, Carmen Lidia Chițescu, Giorgiana Valentina Costea, Daniela Mădălina Anghel, Ana Maria Vlasceanu, Daniela Luiza Baconi

**Affiliations:** 1 Department of Toxicology, Carol Davila University of Medicine and Pharmacy, 37 Dionisie Lupu Street, Sector 2, 20021 Bucharest, Romania; oana.gheorghiu@drd.umfcd.ro (O.R.C.G.); claudia.gutu@umfcd.ro (C.M.G.); madalina.anghel@drd.umfcd.ro (D.M.A.); daniela.baconi@umfcd.ro (D.L.B.); 2 Department of Drug Analysis, Carol Davila University of Medicine and Pharmacy, 37 Dionisie Lupu Street, Sector 2, 20021 Bucharest, Romania; 3 Faculty of Medicine and Pharmacy, “Dunărea de Jos” University of Galați, 32 Eroilor Street, 800119 Galați, Romania; carmen.chitescu@ugal.ro; 4 Faculty of Food Science and Engineering, “Dunărea de Jos” University of Galați, 111 Domnească Street, 800201 Galați, Romania; giorgiana.blaga@ugal.ro

**Keywords:** adulterants, dietary supplements, sildenafil, tadalafil, HPTLC, UHPLC-HRMS-MS

## Abstract

This study proposed a high-performance thin-layer chromatography (HPTLC) screening method to detect phosphodiesterase 5 (PDE-5) inhibitors as possible adulterant agents in various dietary supplements. Chromatographic analysis was performed on silica gel 60F254 plates using a mixture of ethyl acetate:toluene:methanol:ammonia in a volume ratio of 50:30:20:0.5 as a mobile phase. The system provided compact spots and symmetrical peaks of sildenafil and tadalafil with retardation factor values of 0.55 and 0.90, respectively. The analysis of products purchased from the internet or specialized stores demonstrated the presence of sildenafil, tadalafil, or both compounds in 73.3% of products, highlighting inadequacies and inconsistencies in the labeling, as all dietary supplements were declared to be natural. The results were confirmed using ultra-high-performance liquid chromatography coupled with a positive electrospray ionization high-resolution tandem mass spectrometry (UHPLC-HRMS-MS) method. Furthermore, in some samples, vardenafil and various analogs of PDE-5 inhibitors were detected using a non-target HRMS-MS approach. The results of the quantitative analysis revealed similar findings between the two methods, with adulterant quantities found to be similar to or higher than those in approved medicinal products. This study demonstrated that the HPTLC method is a suitable and economical method for screening PDE-5 inhibitors as adulterants in dietary supplements intended for sexual activity enhancement.

## 1. Introduction

*Erectile dysfunction* (ED) is defined as the inability to achieve or maintain an erection during sexual activity. Having multiple causes, this affects almost 30% of men aged over 40 years. The mechanism of the erection includes initiation from the brain or via stimulation of the penis, activation of parasympathetic fibers in the spinal cord, the release of nitric oxide that produces cyclic guanosine monophosphate (cGMP) with muscle relaxation, and increasing intracavernous pressure. The cGMP produced within the process is broken down by the enzyme phosphodiesterase 5 (PDE-5), which re-establishes the basal sympathetic tone [1]. As mentioned before, the causes for this pathology are various: endocrine disease, nerve damage, cardiovascular problems, damage to the penis (trauma), and side effects of medication (beta blockers and thiazides) [1]. Treatment includes PDE-5 inhibitors as the first-line treatment, alprostadil or vasoactive intestinal polypeptide, and phentolamine (intraurethrally) or a penile implant (as the last treatment line). Other treatments include the administration of testosterone [1].

The first PDE-5 inhibitors approved worldwide were sildenafil, tadalafil, and vardenafil [2].

**Figure 1:**
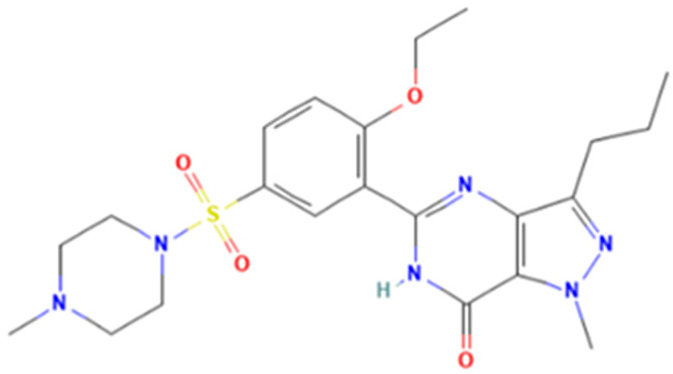
Sildenafil.

*Sildenafil* (5-[2-ethoxy-5-(4-methylpiperazin-1-yl)sulfonylphenyl]-1-methyl-3-propyl-6*H*-pyrazolo[4,3-*d*]pyrimidin-7-one) is a pyrazolopyrimidinone derivative (Figure 1) with vasodilating and potential anti-inflammatory activities as a selective and competitive PDE-5 inhibitor [3]. In Romania, the National Agency for Medicine and Medical Devices of Romania (NAMMDR) lists sildenafil medicinal products of the following therapeutic strengths: 25, 50, and 100 mg. The adverse effects related to sildenafil consumption include headache, flushing, dizziness, dyspepsia, nasal congestion, and altered vision [4,5,6]. Furthermore, disturbed color vision and temporary effects on the electroretinogram (ERG) were reported [7]. Although liver toxicity induced by sildenafil consumption is a very rare event, there have been reported cases over the past 20 years (as the first case of hepatic toxicity of sildenafil was reported in 2003) [8,9]. Most of them were actually related to various aphrodisiac supplements marketed as “natural” or “herbal” remedies for ED, which were found to contain sildenafil or other PDE-5 inhibitors [9]. 

**Figure 2:**
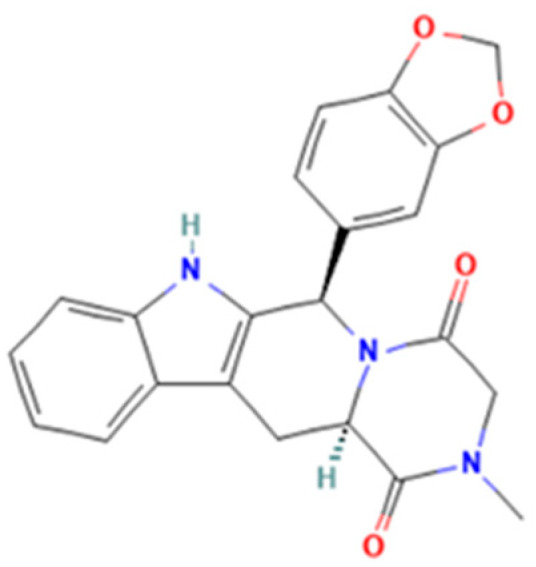
Tadalafil.

*Tadalafil* (2*R*,8*R*)-2-(1,3-benzodioxol-5-yl)-6-methyl-3,6,17-triazatetracyclo [8.7.0.03,8.011,16]heptadeca-1(10),11,13,15-tetraene-4,7-dione) is a pyrazinopyridoindole (Figure 2) that induces prolonged muscle relaxation, vasodilation, and blood engorgement of the corpus cavernosa, resulting in prolonged penile erection. Tadalafil has greater selectivity for PDE-5 and a longer half-life, making it more suitable for chronic once-daily dosing in the treatment of pulmonary arterial hypertension [10]. The medicinal products approved by the National Agency for Medicine and Medical Devices of Romania (NAMMDR) contain 2.5, 5, 10, and 20 mg tadalafil per unit dose. Adverse effects in the case of overdose include headache, dyspepsia, back pain, myalgia, nasopharyngitis, and dizziness [5,6,10,11].

**Figure 3:**
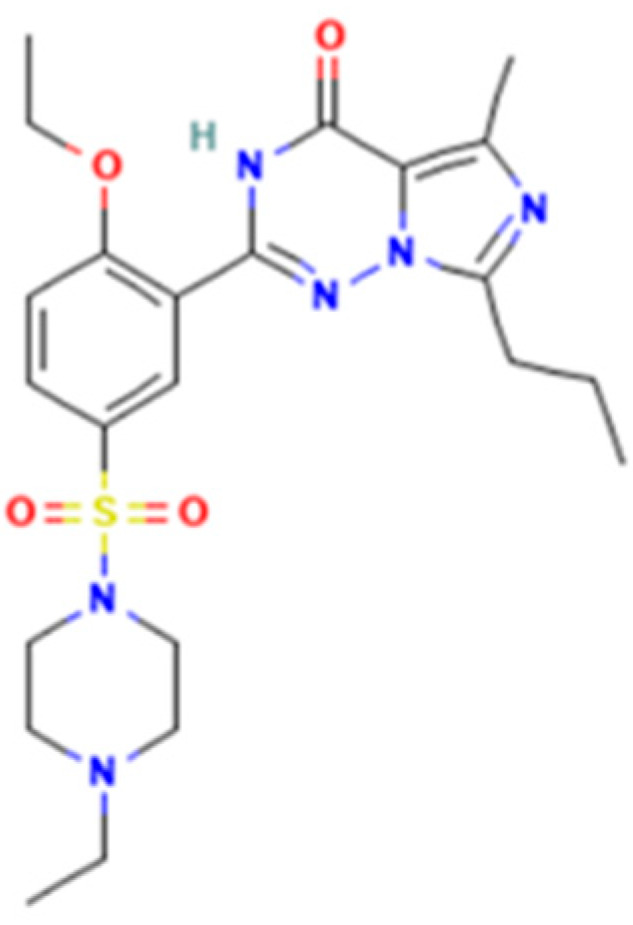
Vardenafil.

*Vardenafil* (2-[2-ethoxy-5-(4-ethylpiperazin-1-yl)sulfonylphenyl]-5-methyl-7-propyl-3*H*-imidazo[5,1-*f*][1,2,4]triazin-4-one) is a benzenesulfonamide derivative (Figure 3) with similar effects on corpus cavernosa as sildenafil and tadalafil [12]. In Romania, medicinal products containing 5, 10, or 20 mg vardenafil per unit dose were approved. Vardenafil was rarely associated with liver injury and serum enzyme elevations [12,13]. The adverse effects include mild headache, dyspepsia, hypotension, dizziness, and minor prolongation of QT [11].

Alongside approved PDE-5 inhibitors medications, herbal dietary supplements are sold worldwide for ED, claiming efficacy through inherent plant constituents and marketed as 100% natural.

Recent animal and human studies suggest that certain plants and plant constituents, such as *Panax gingseng*, *Lepidium meyenii* (maca), *Tribulus terrestris*, *Epimedium* spp., and *Ginkgo biloba* could be efficient as natural aphrodisiacs and have intrinsic PDE-5 inhibition properties [14].

*Panax ginseng* belonging to the family Araliaceae has a reputation as one of the world’s best aphrodisiacs. Its root contains triterpene saponins, such as ginsenosides, as well as essential oil containing polyacetylenes and sesquiterpenes. Ginsenosides were shown to enhance both acetylcholine-induced and transmural-nerve-stimulation-activated relaxation associated with increased tissue cGMP. The ginsenoside-enhanced release of NO from endothelial cells, especially in the corpora cavernosa, was suggested to partly contribute to the aphrodisiac effect of *Panax ginseng* [15]. A systematic review and meta-analysis provided evidence for the effectiveness of *Panax ginseng* (red ginseng) in treating erectile dysfunction [16].

*Lepidium meyenii* (also known as Maca) is a member of the Cruciferae family, and its root and the lower part of the hypocotyl are used for medicinal purposes. The plant contains a complex composition of essential amino acids, polyphenols, phytosterol, macamide, imidazole alkaloids, macaenes (acyclic polyunsaturated oxoacids), and glucosinolates, among others [17]. Studies showed that Maca extract can improve male spermatogenesis and enhance sexual function in animals [18,19]. Oral treatment with Maca improved sperm production and sperm motility in adult men [20]. Administration of Maca tablets (containing 500 mg dehydrated roots) in men was shown to increase sexual desire [21].

*Tribulus terrestris*, which is a flowering plant from the family Zygophyllaceae, contains protodioscin as a chemical constituent and was shown to increase androgenic status, both centrally and peripherally; its administration to humans and animals improves libido and spermatogenesis [15].

*Epimedium* species (horny goat weed) contains icariin as a major bioactive constituent and have been utilized in traditional Chinese medicine for the treatment of erectile dysfunction for many years. Icariin is a flavanol glycoside and a major bioactive constituent of Epimedium, which has an inhibitory effect on all three PDE-5 isoforms [22]. In addition, icariin was found to have testosterone-mimetic properties [23] and to induce nitric oxide synthase expression in corpus cavernosum smooth muscle, resulting in beneficial effects on erectile function [24].

*Ginkgo biloba* is a long-lived, woody, deciduous, and prehistoric tree native to China that contains a variety of pharmacologically active compounds, such as proanthocyanidins, phenolic acids, flavonoid glycosides (kaempferol, myricetin, quercetin), and the terpene lactones. *Ginkgo biloba* has some degree of evidence supporting the idea that it may be helpful for erectile dysfunction. Although the mechanisms responsible for these effects remain poorly understood, it was shown that Ginkgo has the potential to increase blood flow to genitalia [25]. *Ginkgo biloba* extract was demonstrated to improve erectile dysfunction after bilateral cavernous nerve injury in a rat model, suggesting the efficacy of this extract to repair the cavernous nerve and recovery of erectile function after radical prostatectomy [26]. However, a recent systematic review of clinical trials concluded that *Ginkgo biloba* has limited positive effects on sexual function and subsequent trials are needed to confirm its efficacy [27].

Although it was claimed that natural compounds have beneficial effects on erectile dysfunction, adequate clinical evidence to support their use is minimal and these compounds appear to have only a modest effect [28].

Moreover, the claimed efficacy of these products could be the result of intentionally added pharmaceutical ingredients. Therefore, PDE-5 inhibitors are frequently illegally used as adulterants in herbal-declared products for ED that aim to enhance sexual performance [2]. This practice of adulterating food supplements for both potency and losing weight is a common phenomenon worldwide [4]. Moreover, the risk of harm is increased by adding synthetic drugs and various impurities [6].

A survey of the literature revealed more than 50 unapproved structurally modified analogs of PDE-5 inhibitors as adulterants, with sildenafil being the most frequently found, followed by vardenafil and tadalafil [2]. A list of various analogs of sildenafil, tadalafil, and vardenafil is synthesized in Table 1 below [2,29].

Given that the adulteration of dietary supplements is a major problem by putting consumers at risk, a short review of the literature was undertaken in order to identify the analytical methods of extraction of the interest compounds from various herbal products, as well as their determination. The literature survey revealed a wide range of analytical techniques employed for the detection and determination of interest compounds in herbal products, including GC-MS (gas chromatography–mass spectrometry), LC-MS (liquid chromatography–mass spectrometry), HPLC (high-performance liquid chromatography), NMR (nuclear magnetic resonance) spectroscopy, vibrational spectroscopy, LC-FT-ICR-MS (liquid chromatography–Fourier transform ion cyclotron resonance–mass spectrometry), liquid chromatograph–hybrid triple quadrupole linear ion trap mass spectrometer with an information-dependent acquisition, UHPLC-TOF-MS (ultra-high performance liquid chromatography–time of flight–mass spectrometry), IMS (ion mobility spectroscopy), and immunoassay methods [2,5,6,8,30,31]. A brief description of the analyzed methods along with the targeted analyte is described below.

For sildenafil and other types of adulterants, such as tramadol and diazepam, the methods of analysis using HPLC and GC-MS were successfully applied. For the extraction, samples were alkalinized (with 0.1 M borate buffer, pH = 9.2) and dispersive liquid microextraction was performed using methanol and chloroform as the disperser and extraction solvents, respectively. The determination was performed via GC-MS (using an HP5-MS model capillary column cross-linked 5% methylphenyl silicone, 30 m length × 0.25 mm ID × 0.25 μm film thickness) and HPLC (using a C18 column, 250 mm × 4.6 mm, 5 μm particle size, 100 Å pore size, and a mixture of acetonitrile and phosphate buffer 38:62 as the elution solvent in an isocratic elution) [30].

For tadalafil, one study assessed the possible adulteration of dietary supplements using a liquid–liquid extraction (water/CH_2_Cl_2_, alkalinized with 0.1N NaOH to pH = 10) followed by re-crystallization from ethanol. The first step in the analysis involved revealing the presence of a major compound via TLC (thin layer chromatography), which was performed on silica-gel-60-coated F254 aluminum plates using ethyl acetate:water:n-butanol (25:50:100, *v*/*v*/*v*) and chloroform:methanol:diethylamine (9:1:0.1, *v*/*v*/*v*) as mobile phases. The study described the detection of spots with UV light (254 and 366 nm) and via derivatization with Draggendorff and anisaldehyde/sulfuric acid reagents. This was followed by the identification of tadalafil via various spectroscopic techniques, such as mono- and bi-dimensional nuclear magnetic resonance (using dimethylsulfoxide-D6 as the solvent), Fourier-transform infrared (FT-IR) spectroscopy, and mass spectrometry [6]. Quantification of tadalafil was performed via high-performance liquid chromatography–mass spectrometry (HPLC-MS/MS) [6]. The quantification of the target compound was done using a mixed-mode stationary phase HPLC column (hydrophobic alkyl chain with a diol group at the terminus). The mobile phase consisted of a mixture of 0.1% formic acid in MeOH and 0.1% formic acid in water (95:5 *v*/*v*) using an isocratic elution [6].

For vardenafil (and for sildenafil, tadalafil, and other analogs), the literature reveals the importance of applying a tiered approach, combining a bioassay, LC-MS/MS followed by a PDE-Glo bioassay (to select the positive fraction), and further examination with LC–MS/MS and ^1^H-NMR [8]. For the screening with LC full-scan high-resolution MS, an extraction solvent of 1% acetic acid in ACN:H_2_O (80:20, *v*/*v*) was used. For LC-MS/MS (3.0 × 100 mm, 3 µm LC column) analysis, 1% acetic acid in MeOH:H_2_O:ACN 70:20:10 (*v*/*v*) was used as the extraction solvent. The mobile phases consisted of 5.0 mM ammonium formate prepared in ultrapure deionized water (A) and methanol (B), both of which contained 0.1% formic acid, using gradient elution. The PDE-Glo bioassay (Phosphodiesterase Assay Technical Bulletin) involved the use of a kinase glo reagent containing kinase glo substrate and kinase glo buffer and the measurement of luminescence signals in relative light units (RLUs). Active fractions were subsequently analyzed (after solving in ACN:H_2_O (80:20 *v*/*v*)) using time-of-flight mass spectrometry (TOF-MS) with a C18 column 155 × 2.2 mm, 3 µm, as a stationary phase and a mobile phase consisting of ultrapure deionized water and ACN in gradient elution. For ^1^H-NMR, DMSO-D6 was used as the solvent [14].

Other methods were successfully applied for the identification of all three aforementioned PGE-5 inhibitors, namely, sildenafil, tadalafil, and vardenafil. Samples were screened for adulteration using HPTLC (performed on Merck silica gel 60 F254 Premium Purity HPTLC glass plates 20 cm × 10 cm, 0.2 mm) [5,31]. In one method, mass spectrometric analysis of the material derived from individual zones was performed. HPTLC/ESI-MS spectra were directly recorded using the TLC-MS interface coupled to the ESI interface of a single-quadrupole mass spectrometer [31]. Both standard solutions and samples were prepared by dissolving the analytes in methanol. The mobile phase for HPTLC consisted of tert-butyl methyl ether:methanol:ammonia 20:2:1 (*v*/*v*/*v*) [31] or methanol:ethyl acetate 40:80 (*v*/*v*) [5].

Identification of novel sildenafil analogs in an adulterated herbal food supplement (including tadalafil and vardenafil) described the use of LC–MS in ESI negative mode, UV, and NMR spectroscopy [32]. The sample preparation involved a mixture of methylene chloride and 2M NaOH for the extractions and acetonitrile for the reconstitution of the residue. For the LC-DAD analysis, a C18 column (250 mm × 4.6 mm × 5.0 µm) and gradient elution conditions were used (mobile phase A—10 mM ammonium formate and mobile phase B—acetonitrile). For the LC-MS analysis, a C18 column (150 mm × 2.0 mm × 3.0 µm) was used, while for the NMR spectroscopy, dichloromethane was used as the solvent [32].

Another method applicable for sildenafil, tadalafil, and vardenafil consisted of a flow injection tandem mass spectrometry method (FI–MS/MS, which was used for semi-quantification) using the multiple reaction monitoring (MRM) mode. LC-MS/MS was used for further confirmation and quantification [33].

Although there are various methods of analysis available among the chromatographic methods, TLC densitometry has the advantage of easily achieved performance, high accuracy, low limit of detection, and is a low-cost procedure. This method can be successfully used to identify samples of herbal products and supplements both qualitative and quantitative. Qualitative analysis is carried out by comparing the test samples with the standard from the Rf value, UV spectrum, and color [34,35].

The aim of this study was to assess, through a newly developed high-performance thin-layer chromatography (HPTLC)–densitometry method, the accuracy of the apparent active content of herbal food supplements that claim to naturally enhance sexual performance. Therefore, we examined whether the analyzed products were adulterated with active pharmaceutical ingredients of approved pharmaceuticals, such as PDE-5 inhibitors, sildenafil, tadalafil, or other analogs. Additionally, we compared the results obtained by applying the HPTLC method to detect adulterants with those obtained by the more sensitive liquid chromatography coupled with a tandem high-resolution mass spectrometry (LC-HRMS-MS) method.

## 2. Results

### 2.1. HPTLC Method Development

In the first step of the experiments, we used standard solutions to perform a series of tests to select the optimal developing solvent system to separate the analytes and to establish the calibration curves. Different mixtures of organic solvents in various volume compositions and silica gel 60 F 254 chromatographic plates were used.

Initially, a mixture of methanol:ethyl acetate in a volume ratio of 1:2 was used as the mobile phase. The concentrations of standard solutions of sildenafil and tadalafil for the calibration curve were 2 µg, 4 µg, 8 µg, 12 µg, and 16 µg/spot. The measurement of absorbance was performed at a wavelength of 254 nm. The results were as follows: Rf (sildenafil) = 0.47, with maximums of absorption at 240 nm and 306 nm, and Rf (tadalafil) = 0.96, with maximums of absorption at 250 nm and 289 nm. A second analysis using the same experimental conditions was performed for concentrations of standard solutions of sildenafil and tadalafil of 10 µg, 20 µg, 30 µg, 40 µg, and 50 µg/spot, showing similar results: Rf (sildenafil) = 0.49, with maximums of absorption at 240 nm and 306 nm, and Rf (tadalafil) = 0.97, with maximums of absorption at 250 nm and 289 nm.

As previous experiments failed to achieve an adequate separation of sildenafil and tadalafil, a third analysis was performed using a mixture of methanol:ethyl acetate:ammonia with a ratio of 29:60:1 *v*/*v*/*v* as mobile phase and standard solutions of sildenafil and tadalafil at concentrations of 8 µg, 12 µg, 16 µg, 20 µg, and 24 µg/spot. The measurement of absorbance was performed at wavelengths of 254 nm, 289 nm, and 306 nm.

Finally, a mobile phase consisting of a mixture of ethyl acetate:toluene:methanol:ammonia with a ratio of 50:30:20:0.5 *v*/*v*/*v*/*v* was selected, as the best separation of the two compounds was obtained. The concentrations of sildenafil and tadalafil for the calibration curve were 8 µg, 12 µg, 16 µg, 20 µg, and 24 µg/spot, and the measurements of absorbance were performed at wavelengths of 254 nm, 280 nm, and 306 nm. The wavelength was selected based on the maximum absorbance of the UV-VIS spectra for the compounds of interest.

The specificity of the method was confirmed by comparing the Rf values and the spectra of the samples and the standard spots of sildenafil and tadalafil. The spectra showed that there were no interferences and the proposed TLC method was specific and suitable for the determination of sildenafil and tadalafil in the tested herbal supplements. Moreover, the peak purity of adulterants was assessed by comparing the respective densitograms at the peak start, peak middle, and peak end, and the average peak purity index was calculated (Table 2). The peak purity index was above 0.999 (between 0.999360 and 0.999801), suggesting that the adulterants were successfully detected under experimental conditions.

The linearity of the method was evaluated by determining five levels of standard solutions of sildenafil and tadalafil in the range of 8 µg/spot to 24 µg/spot. Regression analysis of the results was used to obtain a linear regression model. The correlation coefficient (R = 0.99) indicated that the method was linear in the range of 8–24 µg/spot for both substances.

The accuracy (as the percent recovery) and precision (as the coefficient of variation) were evaluated using the standard addition method at three concentration levels (80%, 100%, and 120%). For sildenafil, the accuracy ranged from 91.30% to 101.01%, while for tadalafil, it ranged from 93.45% to 100.92%. The accuracy achieved in this study outperformed a previously published HPLC method [36]. Additionally, the good performance of the method was demonstrated by the precision results ranging from 0.22% to 1.08% for sildenafil and 0.73% to 1.32% for tadalafil, all of which fell within the acceptable range of 1–5% for CV values in TLC densitometry [37].

### 2.2. Analysis of Herbal Supplements

#### 2.2.1. Characterization of Analyzed Products and HPTLC Analysis

A total of 15 herbal products used to enhance men’s potency were purchased from the internet or from specialized stores and were analyzed for the presence of adulterating agents, namely, sildenafil and tadalafil. The prices ranged from 0.28 to 9.8 EUR per unit dose. The selection of the analyzed products was based on the specified composition, with most of them containing herbal mixtures, and on the degree of satisfaction declared by the users. The products were marketed as natural and gained popularity by asserting their safety without causing any harmful side effects. All products were formulated as hard capsules, except for sample 12, which was in a tablet dosage form.

A list with various details regarding labeling, as well as the results regarding the presence of adulterants in each analyzed product, is synthesized in Table 2.

The analysis of samples 1, 2, 3, 4, 6, 7, 8, 9, 10, and 14 showed the presence of the undeclared sildenafil ingredient. Sample 6 was positive for both sildenafil and tadalafil, while sample 11 was positive for tadalafil. The analysis of samples 12, 13, and 14 indicated no presence of undeclared PGE-5 inhibitors (Table 2).

**Figure 4:**
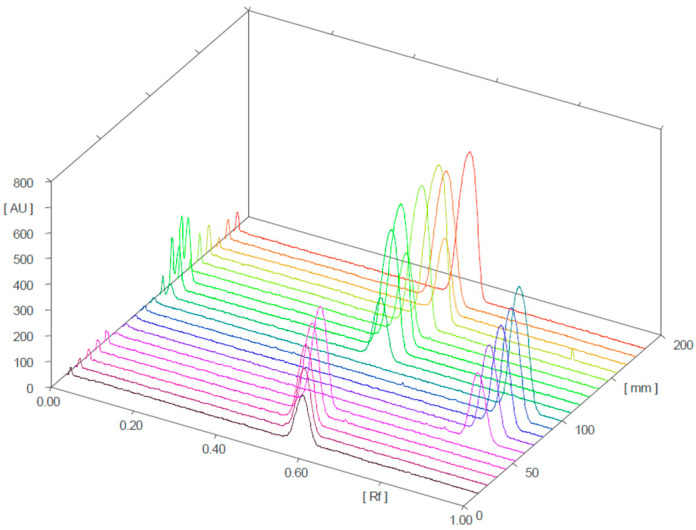
Three-dimensional chromatogram for sildenafil (standard), tadalafil (standard), and samples 1, 2, and 3 (detection at λ = 254 nm; samples 1, 2, and 3 were positive for sildenafil).

**Figure 5:**
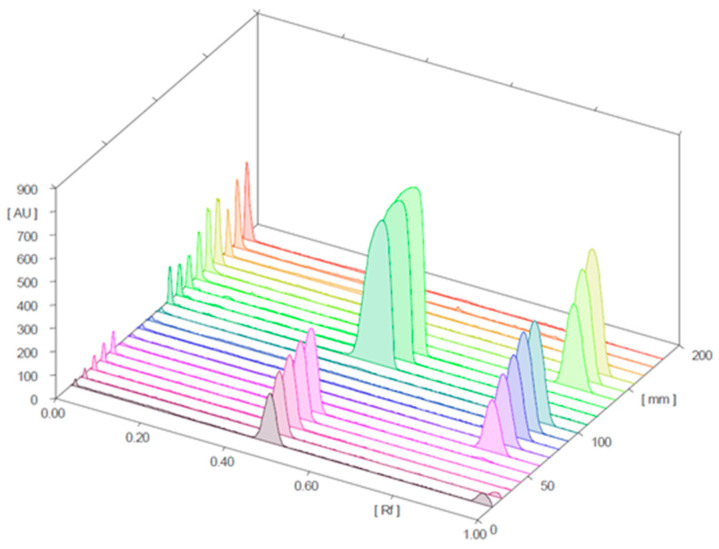
Three-dimensional chromatograms for sildenafil (standard), tadalafil (standard), and samples 10, 11, and 12 (detection at λ = 254 nm; samples 10 and 11 were positive for sildenafil and tadalafil, respectively; sample 12 was negative).

Figure 4 and Figure 5 present the 3D chromatograms obtained from the HPTLC analysis of samples 1, 2, 3, 10, 11, and 12. Each sample was spotted at three concentration levels.

**Figure 6:**
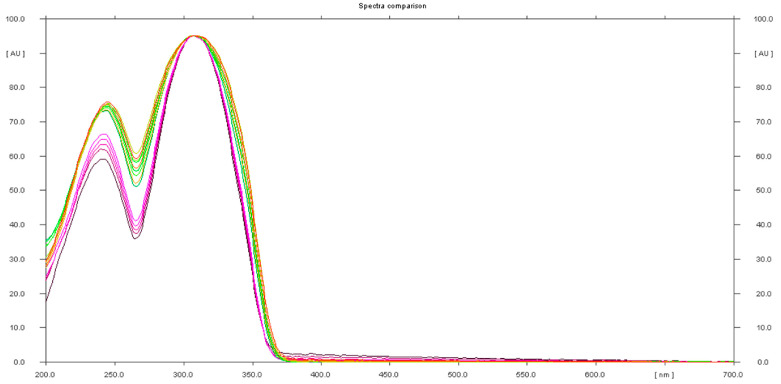
In situ absorbance–reflectance UV spectra of sildenafil standards and samples 1, 2, and 3.

The presence of sildenafil in the herbal samples was confirmed using UV spectra of standards and samples (Figure 6).

The analysis of sample 4 showed the presence of undeclared sildenafil. In addition, an unknown spot was identified at Rf 0.78, similar to sample 5. This spot was identified as caffeine using the UV spectrum from the HPTLC determination (λ_max_ = 275 nm). The result was confirmed via UHPLC-HRMS-MS analysis.

**Figure 7:**
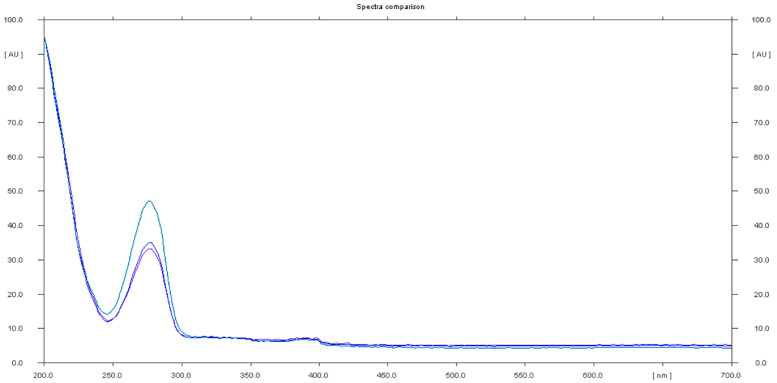
In situ absorbance–reflectance UV spectra of caffeine in sample 5 (Rf = 0.78).

Sample 5 had a negative result for sildenafil identification, but was positive for caffeine, as a spot with Rf 0.78 and a UV spectrum of caffeine was identified (Figure 7).

The analysis of sample 6 showed the presence of both undeclared sildenafil and tadalafil ingredients. Furthermore, an unidentified compound at Rf 0.45 was revealed. This compound had a UV spectrum with maxima at 295 nm and 226 nm.

**Figure 8:**
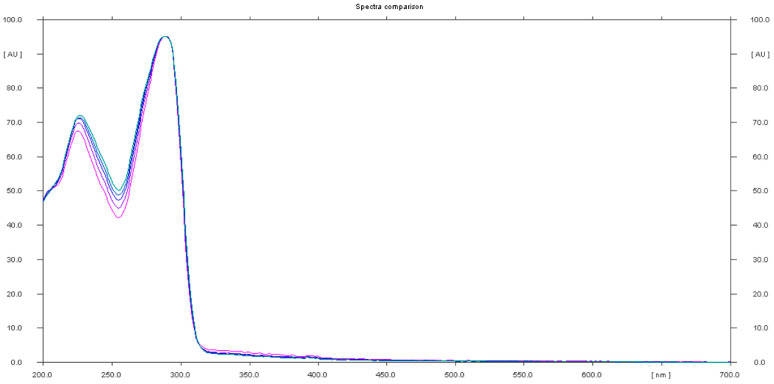
In situ absorbance–reflectance UV spectra of tadalafil standards and sample 11.

The analysis of sample 11 showed the presence of an undeclared tadalafil ingredient. The chromatogram showed a peak at Rf that corresponded to the tadalafil standard, generating the UV spectrum with maxima at λ_1_ = 227 nm and λ_2_ = 289 nm, which are characteristic of the tadalafil standard (Figure 8).

#### 2.2.2. Quantitative Determination of Sildenafil and Tadalafil in Herbal Samples Analyzed Via HPTLC

The quantitative analysis indicated concentrations of sildenafil in a large range from 15 mg/capsule to 116 mg/capsule. For most samples, the amount of sildenafil determined was similar to the concentrations of medicinal products (Table 3). Sildenafil is available in ED medicines with 25 mg, 50 mg, and 100 mg per unit dose. However, the results indicated a high number of samples (five from ten positive sildenafil herbal products) with concentrations similar to the highest therapeutic dose (100 mg/unit dose). Moreover, four products had concentrations that were similar to or higher (23–39 mg/capsule) than the lowest therapeutic dose (25 mg/unit dose). In the case of tadalafil, higher concentrations (about 24 mg and 34 mg/capsule) than the highest therapeutic concentration (20 mg/unit dose) were determined in both positive samples. Tadalafil medicinal products are available in four concentrations: 2.5 mg, 5 mg, 10 mg, and 20 mg/unit dose.

### 2.3. UHPLC-HRMS-MS Analysis

Additional to the HPTLC analysis, high-resolution tandem mass spectrometry (HRMS-MS) was used for confirmatory purposes. An untargeted approach was applied in variable data-independent acquisition (vDIA) mode.

**Figure 9:**
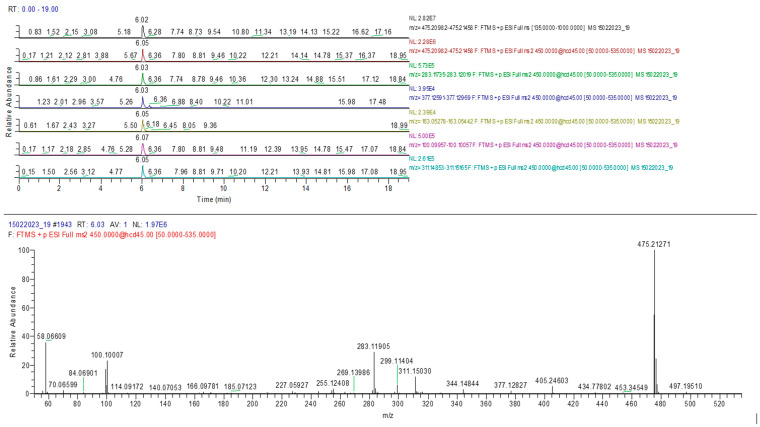
Sildenafil identification in sample 10 (MS-MS).

The results confirmed the presence of the adulterants sildenafil and tadalafil in analyzed samples similar to HPTLC determination. A typical chromatogram and MS spectrum confirming the sildenafil identification are presented in Figure 9.

**Figure 10:**
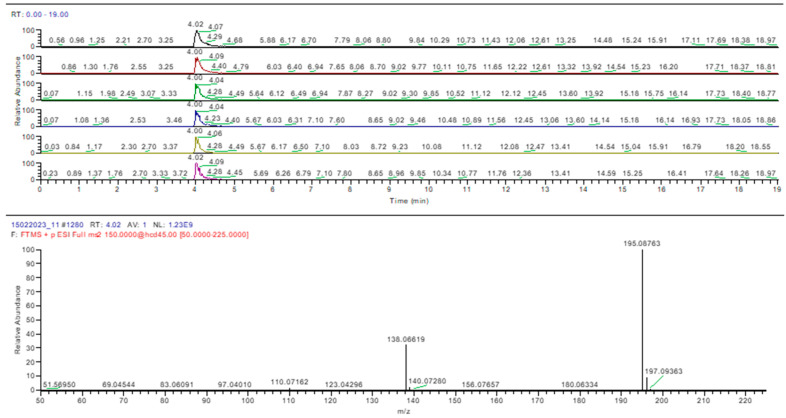
Caffeine identification in sample 4 (MS-MS).

Samples 5, 12, 13, and 15 were negative for sildenafil and tadalafil, as was determined using the HPTLC method. Caffeine was identified in samples 4 and 5, confirming the HPTLC results (Figure 10).

**Figure 11:**
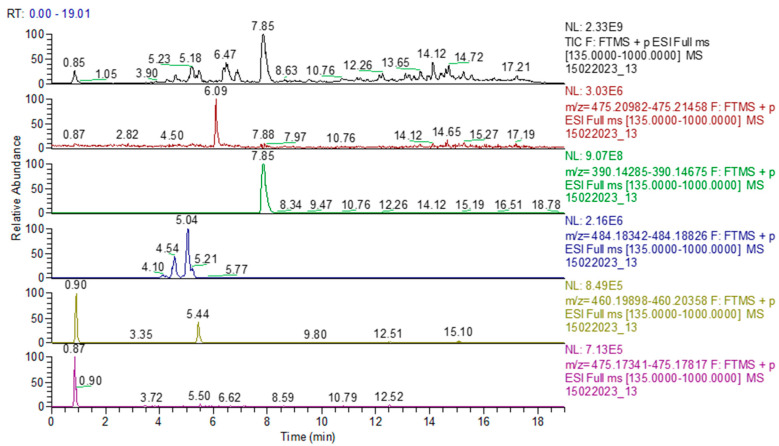
Chromatogram of sample 6: TIC (total ion current) and identification of sildenafil, tadalafil, avanafil, pseudovardenafil, and vardenafil oxopiperazine.

In sample 6, both sildenafil and tadalafil were determined, thus confirming the results obtained using HPTLC (Figure 11). In sample 11, only tadalafil was identified, as determined using HPTLC analysis.

The compounds identified using the UHPLC-HRMS-MS method are presented in Table 4 below.

At least five identification points (one full scan ion in HRMS + one HRMS-MS product ion) were used for the confirmatory analysis according to regulations [38]. Two transitions were used for the ion ratio calculations (in bold in Table 4) for sildenafil and tadalafil. The ion ratios were calculated for all analytes. The ion ratio of the analytes calculated for all samples (Table 5) corresponded to those of the standard solutions at comparable concentrations within a ± 15% relative deviation (lower than the maximum acceptable ± 40% relative deviation).

Two sildenafil analogs, namely, desmethylsildenafil and dimethylsildenafil, were detected in sildenafil-positive samples. The results suggested that these compounds were sildenafil impurities.

Avanafil, vardenafil, and two vardenafil analogs (pseudovardenafil and vardenafil oxopiperazine) were detected in certain samples. The two vardenafil analogs and avanafil were detected in sample 6, while vardenafil was detected in samples 1, 7, 8, and 9 and vardenafil oxopiperazine was detected in samples 4, 6, 7, and 9. Presumptive identification of the compounds without reference standards was based on pattern recognition of the product ions based on a comparison of MS/MS data with reference standards (sildenafil and tadanafil) or in the public MS libraries MassBank (https://massbank.eu/MassBank/) and mzCloudeTM (accessed on 15 February 2023).

Quantitative analysis for sildenafil and tadalafil confirmed the results obtained using the HPTLC method, indicating that the quantity of adulterants exceeded the therapeutic doses in most samples.

## 3. Discussion

A newly developed HPTLC method was applied to screen herbal dietary supplements for enhancing men’s potency to detect possible PDE-5 inhibitors. The analysis of the herbal supplement samples indicated the presence of the PGE-5 inhibitors sildenafil and tadalafil in almost all samples analyzed (11/15). Only three products had negative results (products 12, 13, and 15; Table 2), while 81.81% (9/11) of the samples were positive for sildenafil, 18.18% (2/11) were positive for tadalafil, and 9.09% (1/11) were positive for both sildenafil and tadalafil. The results were confirmed using a sensitive UHPLC-HRMS-MS method.

Our study identified a percentage of 73.33% of adulterated products within the analyzed samples. Although in our study, there was a small number of samples, a high percentage of adulterated products was found, which was consistent with other studies. A survey of the literature investigating the adulteration of herbal products also revealed high percentages of impure products. The study conducted on 91 products revealed a percentage of 81% of adulterated samples with synthetic PDE-5 inhibitors [39]. The most frequently used adulterating agent contained in erectile dysfunction supplements in our study was sildenafil (Table 2), which is a result also supported by other studies’ outcomes [5,30,34,40,41,42]. Similar results were obtained in a study analyzing 20 products using the UPLC method with a PDA detector and a mass spectrometric detector [43].

In sample 4, caffeine was identified along with sildenafil, while in sample 5 only caffeine was detected. The two products had two herbs in common, namely, *Panax ginseng* and *Tribulus terrestris*, but caffeine was not specified in the composition of any of the mentioned species. Therefore, the results suggested that caffeine was added as an adulterant, as it is not declared on the label. Current studies found that caffeine can reduce the prevalence of erectile dysfunction among men with caffeine intake [44]. Preclinical studies indicated that caffeine consumption improved the erectile function of diabetic rats by up-regulating cavernous cGMP [45].

Sample 6 was positive for both sildenafil and tadalafil and this result was confirmed via UHPLC-HRMS-MS analysis. In addition, an unidentified compound with Rf = 0.44 and a UV spectrum with a 296 nm absorption maximum was revealed in product 6. By correlating the results of the two analysis methods and literature data, it is suggested that this compound could be avanafil. It was detected in sample 6 using UHPLC-HRMS-MS. It is worth mentioning that sample 6 was recently reported by the Australian authority to be adulterated with sildenafil and tadalafil, which is a result consistent with our study [46].

Desmethylsildenafil and dimethylsildenafil were detected in sildenafil-positive samples and were absent in sildenafil-negative samples, as well as in the sample with the lowest sildenafil concentration, suggesting that these compounds are sildenafil impurities. Indeed, desmethylsildenafil is listed as sildenafil impurity F according to the Ph.Eur. monograph of sildenafil citrate, and dimethylsildenafil has the same molecular weight (488.22) and a similar structure to sildenafil impurity A; therefore, it is expected to have a similar molecular ion (*m*/*z* = 489.22) and fragments following positive ionization [47].

Other PDE-5 inhibitors, such as avanafil, vardenafil, and two vardenafil analogs (piperidinovardenafil or pseudovardenafil and vardenafil oxopiperazine), were detected in certain samples using the UHPLC-HRMS-MS method. Except for avanafil (possibly present in sample 6 at Rf = 0.44), these compounds were not identified in samples using the HPTLC method. The results suggested that low levels of these compounds were present below the HPTLC limit of detection.

The origin/manufacturer of products was claimed as China (*n* = 9), Romania (*n* = 2), the Netherlands (*n* = 1), and not clearly identified (*n* = 4). All tested samples were labeled as 100% natural; however, except for samples 5, 12, 13, and 15, all the analyzed samples were adulterated with sildenafil, tadalafil, or both. For some samples, labeling was inadequate (e.g., lacking the lot number, expiry date, and/or manufacturer). No samples warned against concomitant nitrate use. PDE-5 inhibitors, such as sildenafil and tadalafil, interact with medications that lower blood pressure, such as nitrates (i.e., nitroglycerin), and may lower blood pressure to dangerous levels. Moreover, no risk of interactions with other pharmaceutical/food supplementary products was listed within the patient leaflet. More than half of the products were manufactured in China (60%). Samples 5 and 14 had the same Romanian manufacturer and were found to be clean (sample 14) or possibly adulterated with caffeine (sample 5). Sample 12 was formulated as tablets, manufactured in the Netherlands, and was found to be negative for adulterants. Regarding sample 13 with an uncertain origin, it was found to be negative for synthetic adulterants. Samples 3, 9, and 14 were produced by the same Chinese manufacturer and their behavior was found to be similar during the analysis. Only sildenafil was detected in these products at a level similar to the highest therapeutic strength of 100 mg (samples 9 and 14) or higher than the lowest therapeutic strength (sample 3). In this study, herbal supplements with uncertain origin or originating from China were found to be adulterated with PDE-5 inhibitors. Therefore, compared with a previous TLC-validated method (using a mixture of methanol:ethyl acetate, 40:80, *v*/*v*, and analyzing 80 samples at 254 nm, lamp D2) [5], similar results were obtained in our study, highlighting a prevalence of adulterated products for sexual enhancement among Asian manufacturers.

Regarding the price of the marketed products, it varied between 0.28 and 9.97 EUR/unit dose. Interestingly, the low-priced supplements were not found to be adulterated (samples 12 and 15).

Quantitative analysis indicated that the amount of sildenafil ranged from 15 mg to 116 mg per hard capsule. For most samples, the concentrations exceeded the lowest approved strength for erectile dysfunction (25 mg) and were similar to or higher than the maximum approved strength (100 mg/unit dose). Tadalafil was quantified in a higher concentration than approved medicinal products (maximum 20 mg/unit dose). Similar outcomes were obtained from other’s researchers’ results [43]. For 18 of the 40 products containing PDE-5 inhibitors, quantitative determination using LC-MS analysis indicated more than 110% of the highest approved strength [37]. Furthermore, the concentrations of PDE-5 inhibitors contained in the samples were above the daily allowable doses [5].

Moreover, besides the analytical testing efforts, tighter regulations that directly address the problem by defining how these products are registered, advertised, and sold are necessary. In order to improve public health and safety, there should be ongoing efforts to research, regulate, and educate the public about sexual enhancement dietary supplements [48].

## 4. Materials and Methods

### 4.1. HPTLC Analysis

#### 4.1.1. Standards

Sildenafil citrate and tadalafil citrate as standard solutions were obtained from Sigma-Aldrich. The standard stock solutions (2.00 mg/mL) of sildenafil and tadalafil were prepared using methanol HPLC (Merck, Darmstadt, Germany). Working solutions of various concentrations were prepared via dilution in the same solvent and were used to obtain the calibration curve.

#### 4.1.2. Solvents

All organic solvents used in this study were of analytical grade. Methanol, ethyl acetate, ammonia, and toluene were purchased from Merck, Germany.

#### 4.1.3. Chromatographic Parameters

Chromatography was performed on pre-coated silica gel F254 glass-back plates (size 20.0 cm × 20.0 cm) obtained from E. Merck KgaA. The plates were used as obtained from the manufacturer without any pretreatment; a Linomat 5 (Camag, Muttenz, Switzerland) equipped with 100 µL syringe was used for the semi-automatic sample application (with a spot of diameter approx. 6–7 mm). The Linomat 5 application parameters were as follows: inert gas nitrogen (as the spray gas), methanol (as the sample solvent), 150 nL/s (dosage speed), and a predosage volume of 0.2 µL.

The mobile phase used was ethyl acetate:toluene:methanol:ammonia in a volume ratio of 50:30:20:0.5. The distance from the lower edge was 20 mm, the distance from the side was 15 mm, and the track distance was 11.4 mm.

Ascending development was performed in a Camag twin-trough chamber (for 20 cm × 20 cm plates) after at least 30 min of saturation; the mobile phase migration distance in all experiments was 10 cm. After being air-dried, the plates were scanned in the TLC scanner. Densitometric scanning of the obtained spots was performed using a Linomat 5 (Camag) operating in the absorbance mode and controlled using WinCATS software version 1.4.4. Deuterium and wolfram lamps were used as the radiation source. The chromatographic plates were scanned with slit dimensions of 4.00 × 0.30 mm and a scanning speed of 20 mm/s. Densitometric analysis of the chromatograms was performed at 254 nm. Additional analysis was performed at wavelengths of 240 nm, 289 nm, and 306 nm. The data resolution speed was 100 µm/step.

#### 4.1.4. Calibration Curve for HPTLC

For the calibration curve, various concentrations of sildenafil and tadalafil in methanol were used as follows: 8 µg, 12 µg, 16 µg, 20 µg, and 24 µg. The peak area and the amount of sildenafil/tadalafil were identified to plot a calibration plot.

### 4.2. Samples Preparation/Extraction

All food supplementary samples, except for sample 12, were presented as hard capsules. The extraction of the content of one capsule or a triturated tablet was performed with 10 mL methanol HPLC via sonication for 10 min. Three units of each sample were evaluated and the average amount of adulterants was assayed.

### 4.3. UHPLC-HRMS-MS Analysis

#### 4.3.1. LC Parameters

A Thermo Scientific Dionex Ultimate 3000 UHPLC (Thermo Fisher Scientific, Waltham, MA, USA) was used for the analysis. Chromatographic separation was achieved using an Accucore U-HPLC Column C18 (150 × 2.1 mm, 2.6 µm; Thermo Scientific) at a flow rate of 0.4 mL/min. The mobile phase consisted of water with formic acid at 250 µL/L (A) and methanol with formic acid at 250 µL/L (B). A 19 min gradient was used. The step gradient was as follows: 0–1 min 95% A, 1–4.5 min linear increase to 40% B, 4.5–15 min linear increase to 100% B and hold for 2 min, 17–17.5 min decrease to 0% B, and 17.5–19 min 95% A. The injection volume was set to a 15 μL HESI (heated electrospray) ion source and was used for the ionization in positive mode. The HESI parameters were optimized as follows: sheath gas flow rate 40 unit, aux. gas unit flow rate 10, capillary temperature 240 °C, aux gas heater temperature 350 °C, spray voltage 2800 V, and S lens RF level 50.

#### 4.3.2. MS Parameters

Detection of the target compounds was performed using a Q-Exactive mass spectrometer (Thermo Fisher Scientific, Waltham, MA, USA). A total of five scan events were combined in vDIA mode: one full scan event at a resolving power of 70,000 FWHM at *m*/*z* 200 and four MS-MS events at a resolving power of 35,000 FWHM. In the MS2 scan events, the precursor ion ranges of *m*/*z* 95–205, 195–305, 295–405, and 395–800 were consecutively selected, fragmented in an HCD cell, and measured in four separate scans. Nitrogen was used as the collision, sheath, and auxiliary gas at 11–48 arbitrary units flow rates. The energy of the collision-induced dissociation cell was varied in the 30–60 eV range. Data were acquired and analyzed with the Quan/Qual Browser Termo Xcalibur software package version 4.1 (Thermo Fisher). The precursor ions were filtered using the quadrupole, which operated at an isolation window of *m*/*z* 2. The mass tolerance window was set to 5 ppm for the two analysis modes. Confirmatory analysis was based on accurate mass measurements and pattern recognition of the product ions based on a comparison of MS/MS data with reference standards (sildenafil and tadalafil) or in public MS libraries as MassBank and mzCloudeTM for tentative identification of the compounds without reference standards.

#### 4.3.3. Calibration Curve for UHPLC-HRMS-MS

For the sildenafil and tadalafil, calibrations were carried out in the 50–1750 μg/L concentration range using a serial dilution of the 1 mg/L standard mix. The R^2^ coefficient for all standards was higher than 0.99, showing good linearity.

## 5. Conclusions

A straightforward HPTLC procedure was developed for the determination of sildenafil and tadalafil as adulterants in various herbal supplements used for enhancing men’s potency. The qualitative and quantitative results obtained using the HPTLC method were confirmed by applying UHPLC-HRMS-MS. Our study revealed a disturbing trend: a significant proportion of dietary supplements were adulterated with PDE-5 inhibitors, and most samples from Asian countries were found to be contaminated. Alarmingly, none of the products listed the adulterants on their labels, thereby putting consumers at risk. The quantitative analysis indicated that the adulterants were frequently added in concentrations similar to or higher than the highest approved strength, further raising health concerns. Compared with the sensitive and sophisticated UHPLC-HRMS-MS, the HPTLC method was simple, economical, time-saving, and could be successfully applied as a routine control test for adulterating substances detection in various food supplementary products destined to enhance sexual activity.

In addition to the analytical testing efforts, there is a need for tighter regulations regarding the registration, advertising, and commercialization of these products.

## Figures and Tables

**Table 1:** PGE-5 inhibitors and their analogs.

PGE-5 Inhibitor	PGE-5 Inhibitor Analogs
Sildenafil	^1^ *N*-desmethylsildenafil, ^1^ homosildenafil, ^1^ alidenafil, ^1^ benzylsildenafil, ^1^ hydroxyhomosildenafil, ^1^ norneosildenafil, ^1^ descarbonsildenafil, ^1^ cyclopentynafil, ^2^ propoxyphenyl sildenafil, ^2^ ropoxyphenyl hydroxyhomosildenafil, ^2^ propoxyphenyl alidenafil, ^2^ mirodenafil, ^2^ udenafil, ^3^ acetildenafil, ^3^ noracetidenafil, ^3^ hydroxyacetildenafil, ^3^ oxohongdenafil, ^3^ isopiperazinonafil, ^3^ nitrodenafil, ^3^ dimethylacetildenafil, ^3^ dioxiacetildenafil, ^3^ piperidino acetildenafil, ^3^ cinnamyldenafil, ^3^ hydroxychlorodenafil
Tadalafil	Nortadalafil, (−)-*trans*-tadalafil, aminotadalafil, *SR*-amino-tadalafil, (+)-*trans*-aminotadalafil, acetaminotadalafil, hydroxypropylnortadalafil, *N*-buthylnortadalafil, *N*-octylnortadalafil, chloropretadalafil
Vardenafil	^4^ *N*-desmethylvardenafil, ^4^ hydroxyvardenafil, ^4^ pipeirinovardenafil, ^4^ pseudovardenafil, ^4^ piperidenafil, ^5^ hydroxythiovardenafil, ^6^ acethylvardenafil, ^6^ norneovardemafil, ^6^ imidazosagatriazinone, desulfovadenafil

^1^ Pyrazolo pyrimidine-7-one, ethoxyphenyl, and sulfonamide moieties; ^2^ pyrazolo pyrimidine-7-one, propoxyphenyl, and sulfonamide moieties; ^3^ pyrazolo pyrimidine-7-one and ethoxyphenyl moieties without a sulfonamide moiety; ^4^ imidazosagatriazinone and sulfonamide moieties; ^5^ imidazosagatriazine-thione andsulfonamide moieties; ^6^ other related compounds.

**Table 2:** Products sampled to highlight labeling inconsistencies and inadequacies, as well as the presence of adulterants—results of HPTLC analysis.

Product Sample No./ Dosage Form	Manufacturer (Country)	Price (EUR/Unit Dose)	Description	Lot Expiry Date	Results: Labeling Inconsistencies/ Inadequacies	Presence of PDE-5 Inhibitor	Peak Purity Index
1/ hard capsules	China	1.00	100% natural product: *Eleutherococcus senticosus*, *Poligonum multiflorum*	1 March 2026	Not a 100% natural product	Sildenafil	0.999687
2/ hard capsules	China	3.5	100% natural product: *Lycium barbarum*, *Cinnamomum cassia*, *Rhodiola rosea*, *Panax ginseng*, *Allium porum*, *Ginkgo biloba*, vitamin D	October 2025	Not a 100% natural product	Sildenafil	0.999701
3/ hard capsules	China	6.7	100% natural product: *Epimedium brevicornum*, *Panax ginseng*, *Rhodiola rosea*, *Cinnamomum zeylancium*	16 March 2024	Not a 100% natural product	Sildenafil	0.999801
4/ hard capsules	Uncertain	3.95	100% natural product: *Panax ginseng*, *Tribulus terrestris*, *Serenoa repens*, *Zingiber officinale*	September 2025	Not a 100% natural product	Sildenafil Caffeine	0.999630 0.999232
5/ hard capsules	Romania	0.28	“Active principles from natural sources”: l-arginine, royal jelly, *Panax ginseng*, *Lepidium meyenii* (Maca root extract), *Tribulus terrestris,* niacin	May 2024	Possibly adulterated	Caffeine	0.999369
6/ hard capsules	Uncertain	6.33	“100% Safe herbal alternative”: l-arginine, magnesium, vitamin C, Maca, ginseng root, saw palmetto (*Serenoa repens*), fenugreek (*Trigonella foenum*), l-selenomethionine, vitamin B5, zinc, vitamin B12	-	Not a 100% natural product	Sildenafil, Tadalafil, Unidentified substance with Rf 0.4	0.999499 0.999623
7/ hard capsules	China	8.66	100% natural product, plant extract: *Panax Ginseng*, *Cinnamonum cassia*, *Apium graveolens*, *Brassica juncea*	9 May 2024	Not a 100% natural product	Sildenafil	0.999730
8/ hard capsules	China	6.00	100% natural product: *Epidemium brevicornum*, *Morinda officinalis*, *Cynomorium songoricum*, *Lycium barbarum*, *Panax ginseng*, *Poria cocos* mushroom, *Dioscorea opposite*, *Calendula officinalis*	1 May 2025	Not a 100% natural product	Sildenafil	0.999659
9/ hard capsules	China	9.80	100% natural product: *Epimedium brevicornum*, *Tribulus terrestris*, *Panax ginseng*, *Rhodiola rosea*, *Lycium chinense*, *Cinnamomum cassia*	17 April 2023	Not a 100% natural product	Sildenafil	0.999710
10/ hard capsules	China	5.72	100% natural product: *Rehmannia glutinosa*, *Panax ginseng*, *Dioscorea opposita*, *Lycium barbarum*, *Alpinia oxyphylla*, *Perenniporia fraxinea*, *Cinnamomum zeylanicum*	18 November 2024	Not a 100% natural product	Sildenafil	0.999373
11/ hard capsules	Uncertain	8.28	100% Natural product: *Gingko Biloba*, *Panax ginseng*, *Cinnamomum zeylancium*, *Poria Cocos* mushroom, *Codonopsis Pilosus*, *Ligusticum* root, *Angelica archangelica*, *Glycyrrhiza glabra*	-	Not a 100% natural product	Tadalafil	0.999669
12/ tablets	The Netherlands	1.60	100% natural product: D-biotin, vitamin B6, vitamin B1, vitamin B12, magnesium	February 2025	Clean	Negative	-
13/ hard capsules	Uncertain	4.40	100% Natural product: *Lycium barbarum*, *Cinnamomum Cassia*, *Codonompsis pilosula*, *Angelica archangelica*, *Panax Ginseng*, *Poria Cocos* mushroom, *Lingusticum porteri*, *Glycyrrhiza Glabra*	February 2025	Clean	Negative	-
14/ hard capsules	China	3.70	100% natural product: *Epimedium brevicornum*, *Tribulus terrestris*, *Panax Ginseng*, *Rhodiola Rosea* roots, *Lycium Chinese*, *Cinnamomum Cassia*	31 March 2025	Not a 100% natural product	Sildenafil	0.999679
15/ hard capsules	Romania	0.34	100% natural product: *Tribulus terrestris*, *Poligonum multiflorum*, *Lepidium meyenii* (Maca extract), *Heracleum sphondylium*, *Turnera difussa*, *Lycium barbarum* (Goji extract), *Ptychopetalum olacoides*, Indian ginseng/Ashwagandha (*Withania somnifera*), Siberian ginseng (*Eleutherococcus senticosus*)	July 2025	Clean	Negative	-

**Table 3:** Concentrations of adulterants (sildenafil and tadalafil) in the analyzed samples.

Adulterant	Number of Samples with Concentration (mg/Capsule)		
	Lower than Therapeutic Concentration	Similar to Therapeutic Concentration	Higher than Therapeutic Concentration
Sildenafil	1 (sample 6)	8 (samples 1, 2, 3, 4, 7, 9, 10, and 14)	1 (sample 8)
Tadalafil	-	-	2 (samples 6 and 11)

**Table 4:** The compounds identified using UHPLC-HRMS-MS with structures that were confirmed via comparison with reference standards (in bold in the table) or were presumed based on high-accuracy analysis of protonated precursors and fragment ions of specific components.

Compound Name	R.T. (min)	Molecular Formula	Exact Mass (*m*/*z*)	Error (ppm)	Adduct Ion (*m*/*z*)	MS^2^ Fragments (*m*/*z*)
**Sildenafil**	6.02	C_22_H_30_N_6_O_4_S	474.20492	0.19	475.21220	377.1278, **311.1500**, **283.1187**, 163.0536, 100.0995
**Tadalafil**	7.85	C_22_H_19_N_3_O_4_	389.13756	0.40	390.14483	**268.107**, **262.0864**, 240.1128, 162.0864,
Desmethylsildenafil	5.17	C_21_H_28_N_6_O_4_S	460.18927	0.83	461.19655	197.0704, 135.0440
Vardenafil	5.20	C_23_H_32_N_6_O_4_S	488.22057	−1.02	489.22783	377.1278, 283.1187, 87.0760
Dimethylsildenafil	6.70	C_23_H_32_N_6_O_4_S	488.22057	0.56	489.22783	376.1074, 312.1581, 169.0968, 72.0808
Pseudovardenafil	0.90	C_22_H_29_N_5_O_4_S	459.19403	0.58	460.20128	377.1278, 311.1503, 113.1073, 99.0917
Vardenafil oxopiperazine	0.87	C_21_H_26_N_6_O_5_S	474.16854	−0.68	475.17579	245.0506, 169.0967
Avanafil	5.06	C_23_H_26_ClN_7_O_3_	483.17856	1.11	484.18584	113.0084
Caffeine	4.02	C_8_H_10_N_4_O_2_	194.08038	−0.66	195.08765	344.1478, 299.1138, 169.0971

**Table 5:** Confirmation of the peak’s identity based on the ratio of the specific fragments.

Product Sample No.	Adulterant Identified	Specific Fragments (*m*/*z*)	Ion Ratio *
1	Sildenafil	311.1500; 283.1187	42.195
2	Sildenafil	311.1500; 283.1187	43.841
3	Sildenafil	311.1500; 283.1187	44.911
4	Sildenafil	311.1500; 283.1187	44.197
6	Sildenafil Tadalafil	311.1500; 283.1187 268.107; 262.0864	40.943 82.265
7	Sildenafil	311.1500; 283.1187	43.057
8	Sildenafil	311.1500; 283.1187	42.857
9	Sildenafil	311.1500; 283.1187	42.944
10	Sildenafil	311.1500; 283.1187	43.723
11	Tadalafil	268.107; 262.0864	92.006
14	Sildenafil	311.1500; 283.1187	43.536

* Min limit = 34.629 and max limit = 46.851 for sildenafil, min limit = 78.729 and max limit = 106.516 for tadalafil.

## Data Availability

The data presented in this study are available on request from the corresponding author.
